# Non-invasive prediction of maca powder adulteration using a pocket-sized spectrophotometer and machine learning techniques

**DOI:** 10.1038/s41598-024-61220-1

**Published:** 2024-05-07

**Authors:** John-Lewis Zinia Zaukuu, Zeenatu Suglo Adams, Nana Ama Donkor-Boateng, Eric Tetteh Mensah, Donald Bimpong, Lois Adofowaa Amponsah

**Affiliations:** 1 Department of Food Science and Technology, Kwame Nkrumah University of Science and Technology, Kumasi, Ghana; 2 Department of Food Science and Technology, Ho Technical University, Ho, Volta Region Ghana; 3 Department of Hospitality Management, Takoradi Technical University, Takoradi, Western Region Ghana

**Keywords:** Adulteration, Chemometrics, Maca, Near infrared, Spectra-preprocessing, Characterization and analytical techniques, Surface patterning, Imaging and sensing

## Abstract

Discriminating different cultivars of maca powder (MP) and detecting their authenticity after adulteration with potent adulterants such as maize and soy flour is a challenge that has not been studied with non-invasive techniques such as near infrared spectroscopy (NIRS). This study developed models to rapidly classify and predict 0, 10, 20, 30, 40, and 50% w/w of soybean and maize flour in red, black and yellow maca cultivars using a handheld spectrophotometer and chemometrics. Soy and maize adulteration of yellow MP was classified with better accuracy than in red MP, suggesting that red MP may be a more susceptible target for adulteration. Soy flour was discovered to be a more potent adulterant compared to maize flour. Using 18 different pretreatments, MP could be authenticated with R^2^_CV_ in the range 0.91–0.95, RMSE_CV_ 6.81–9.16 g/,100 g and RPD 3.45–4.60. The results show the potential of NIRS for monitoring Maca quality.

## Introduction

“*Lepidium meyenii*”, known as maca or Peruvian ginseng, is an edible herbaceous biennial plant of the family Brassicaceae that is native to South America in the high Andes mountains of Peru and Bolivia (Leon,1964). Maca is a natural nutraceutical product regarded as a “superfood”^1^. According to the Food and Agriculture Organization of the United Nations, the term “superfoods” was coined in 2005 by the food and beverage industry to designate a variety of fruits and vegetables regarded to bestow important and vital components to human health and nutrition^2^. There are three distinct hypocotyl colors for Maca, namely red, yellow, and black which are also sometimes referred to as their cultivars^3^.

The effectiveness and safety of consuming these maca cultivars have been evaluated in numerous clinical studies with the majority of these studies concentrating on how Maca affected sperm count, sexual behavior^4^ and reduce dosteoarthritis pain and stiffness^5^. As the demand for Maca on the global market slowly rises due to its benefits, dishonest individuals have started adopting inexpensive substitutes to either adulterate or fabricate maca and boost their profit.

Existing techniques to detect maca origin and adulteration are time-consuming and costly. Maca adulteration has been investigated with DNA-barcoding approach based on the Internal transcribed spacer (ITS)^6^. Chemical profiling analysis of Maca using ultra-high- performance liquid chromatography (UHPLC),electrospray ionization mass spectrometry (ESI-Orbitrap) coupled with UHPLC-ESI-QqQ MS and the neuroprotective study on its active ingredients has also been reported^7^. Reported techniques for maca cultivar discrimination are the ESI^8^, the stable isotope ratio and mineral elemental fingerprints^9^, liquid chromatography-ultraviolet detection -tandem mass spectrometry (LC-UV–MS/MS)^10^. In recent years, quick techniques for food adulteration and authenticity are crucial^11,12^.

Spectroscopy is a science of light interaction (absorbance/reflectance) with an analyte across the electromagnetic spectrum. With just one test, spectroscopic techniques can gather a lot of data quickly and affordably. Additionally, spectroscopic methods only need little to no sample preparation^11,13,14^. In some instance, these spectroscopic techniques are used to identify the specific entities; however, fingerprints can also be established as a more efficient screening technique. Most of these techniques are liaised with chemometric tools such as Principal component analysis (PCA), hierarchical cluster analysis (HCA), linear discriminant analysis (LDA), soft independent modeling by class analogy (SIMCA), and partial least squares regression (PLSR) to improve the selection of the most relevant instrumental data output^15,16^. NIRS in tandem with chemometric techniques has been used to successfully identify different adulterations based on compositional variations and disparity in chemical functional groups in several studies including honey, oil and ground powders^12,15^.

Different maca samples adulterated with turnip and radish powder individually at different percentages (5–95%) could be detected with near infrared spectroscopy (NIRS)^17^. In another study, pure maca powder mixed with rice flour and rice bran at proportions of 25%, 50%, and 75% could be classified with good accuracies using NIRS^18^. Even the fourier transform near infrared spectroscopy (FTIR) analytical method has been used to detect sucrose in Maca powder^19^.

A major gap in all of these studies Is that only benchtop instruments were used and adulteration of multiple maca cultivars were not tested. The emergence of handheld devices can cut analytical cost and avoid analytical technicalities. It also presents the advantage of remote analysis or on-site analysis. In addition, novel suspicious adulterants such as soybean flour and maize flour which bear a striking physical resemblance to maca powder were not tested. Lastly, multiple spectra preprocessing techniques which are known to increase model robustness were not test. There is also no reported study of using NIRS to discriminate maca cultivars.

Thus, the objective of this work was to create models utilizing a handheld near-infrared spectrophotometer to quickly classify and predict low to high amounts of soybean and maize flour in three different cultivars of maca powder and also to develop models to discriminated the different cultivars irrespective adulteration.

## Materials and methods

### Sample acquisition

Authentic Maca (*Lepidium meyenii)* and adulterants were purchased from reliable vendors (health stores) in the Greater Accra Region of Ghana in accordance with the International Union for Conservation of Nature (IUCN) policy statement on research involving species at risk of extinction and also, according to the Kwame Nkrumah University of Science and Technology Research (KNUST POLICY 0003) and Ethics policy (KNUST POLICY 0007). Three major types of Maca (cultivar) powder were purchased: yellow Maca (YM), black Maca (BM), and red Maca (RM). Soybean powder and maize powder were also acquired at a reputable market in the Greater Accra region of Ghana and used as adulterants. The samples were kept in a low-density polyethylene (LDPE) plastic ziplock bag and transported aseptically to the laboratory to be used for the study.

### Sample preparation

Artificial adulteration of Maca powder was performed in the laboratory to mimic the suspected practice on the market. For this, each Maca type in its powdered form was respectively mixed with the adulterants (soy and maize) in powdered form (equal particle size) at six different concentrations: 0, 10, 20, 30, 40, and 50% w/w of the respective adulterant. Each concentration was prepared in triplicate and thoroughly homogenized to yield a total of 108 samples (6 adulterant concentrations × 2 adulterants (soy and maize) × 3 Maca types (Red, Black and Yellow Maca) × 3 (triplicate samples)). The samples were labeled for easy identification while scanning. Soy and maize powder were milled and sieved to have the sample particle size as maca powder before NIRS analyses. This was to ensure effective homogenization of the mixtures and reduce the influence of no-homogenous scanning surfaces that could lead to additive or multiplicative effects on the spectra.

### NIRS measurements

All 108 samples (10 g each) were scanned through the low-density polyethylene (LDPE) zip-lock bags using the handheld DLP NIRScan Nano instrument (Texas Instruments, Dallas, TX, USA). The instrument has a wavelength range of 900–1700 nm and a spectral resolution of 3 nm. Three consecutive spectral measurements were taken for each sample resulting in a total of 324 spectra. The entire spectrum capture process was done at ambient temperature. For each sample, the powder was gathered at one point of the plastic (lower right) before spectra collection as demonstrated by Zaukuu et al. (2020). This was to reduce the influence of no-homogenous scanning surfaces that could lead to additive or multiplicative effects on the spectra.

#### Spectral analysis

The spectra from the NIRS scan were first preprocessed with a Savitzky-Golay smoothing filter to reduce the noise additive effect of the collected spectra before performing a principal component analysis (PCA). PCA was employed to visualize, detect and remove outliers from all the samples. It was also used to reduce dimension while preserving the relevant information.

#### Linear discriminant analysis (LDA)

Linear discriminant analysis (LDA) was then used to develop models to classify the different concentrations based on the type of Maca powder that was being adulterated. In total eight different classification models were developed. The first model was developed to classify the different types of Maca powders in their pure state while the second was to classify the different types of Maca in their adulterated state. This was to ascertain the possibility of varietal differences irrespective of adulteration.

Three models were developed next to classify 0, 10, 20, 30, 40, and 50% w/w soy in yellow, black, and red Maca powders respectively, followed by another three models, which were developed to classify 0, 10, 20, 30, 40, and 50% w/w maize powder in yellow, black and red Maca powders respectively.

By splitting the data into two categories—training and validation—the predictive value of each LDA model was assessed. The first and second replicates, which together made up two-thirds of the data, were represented in the training set by their spectra. The validation set was built using the spectra of the third replication. Calibration and validation set’' replicates were switched out three times during the data splitting phase.

The statistical parameters used to evaluate the performance of the LDA models were the recognition accuracy (%) and prediction accuracy (%). Recognition accuracy (%), represents the accuracy of calibration, whereas prediction accuracy (%), represents the accuracy of cross-validation (%). These were assessed through confusion matrices where, columns represented the actual class membership and the rows represented the predicted class membership. Other parameters used to evaluate the performance of the developed LDA models were the sensitivity, specificity and precision^20^ calculated after cross-validation as followed:

$$Sensitivity \, = \, True \, positives \, / \, \left( {True \, positives \, + False \, negative} \right)$$

$$Specificity \, = \, True \, negative/ \, \left( {True \, negative \, + \, False \, negative} \right)$$

$$Precision \, = \, True \, positives/ \, \left( {True \, positives \, + \, False \, positives} \right)$$

The sensitivity of the test was defined as its ability to determine the true (correct) classes, whereas, specificity refers to the ability to correctly determine the false (incorrect classes). Precision referred to the closeness of two or more measurements to each other. Their values were reported in percentages (%).

#### Partial least square regression (PLSR)

The potential of NIRS to predict concentrations of pure Maca in both the pure and adulterated samples was tested using the PLSR. For the development of the models, 18 different preprocessing techniques were tested using a combination of Savitzky-Golay pretreatment, derivation, standard normal variate, multiplicative scatter correction, and detrending as shown in Table S1.

With leave-one-sample-out cross-validations, all three repeats of each sampl’'s nine spectra were excluded from the validation procedure, allowing researchers to assess the predictive significance of all the PLSR models outlined. The coefficient of determination (R^2^_C_), root mean square error of calibration (RMSE_C_), and the ratio of prediction to deviation (RPD) were the statistical variables used to assess the effectiveness of the PLS regression models (RMSE_CV_, R^2^_CV_). In order to avoid overfitting, the models, the ideal number of latent variables for each model was calculated based on the least RMSE_C_ and RMSE_CV_ values.

Using only the best preprocessing method in the developed models, limit of detection minimum value (LOD), limit of detection maximum value (LODmax), limit of quantification minimum value (LOQ) and sensitivity were calculated through the partial least-squares (PLS) methods according to the International Union of Pure and Applied Chemistry (IUPAC) approach described by Allegrini and Olivieri^21^:

$$LOD = 3.3 \left[ {SEN - var \left( x \right) + h0min \,SEN - var \left( x \right) + h0min \,var \left( {ycal} \right)} \right]^{1/2}$$

$$LOD = 3.3 \left[ {SEN - var \left( x \right) + h0max \,SEN - var \left( x \right) + h0max \,var \left( {ycal} \right)} \right]^{1/2}$$

where, SEN is the sensitivity (inverse of the length of the regression coefficient), var (x) is the variance of the instrument signals. h0min/max is the minimum/maximum distance between a hyperplane for the calibration set, representing the scores of the samples for which the analyte of interest is absent and the center of a normalized calibration score space. Var (ycal) is the variance in the calibration concentrations. LOD correspond to the calibration samples with the lowest and largest extrapolated leverages to zero analyte concentration^22^ Click or tap here to enter text. LOQ was obtained by multiplying the LOD values with a factor value of three (Allegrini and Olivieri, 2014). LOD, LOQ and Sensitivity values were calculated in MATLAB (version 2022b) and used to further evaluate the performance of the models for predicting the parameters of interest. All chemometric data was analyzed with R version R 3.3.0 + (Aquap2 package).

### Ethical approval

Ethics approval was not required for the collection of plant samples according to the Kwame Nkrumah University of Science and Technology Research (KNUST POLICY 0003) and Ethics policy (KNUST POLICY 0007), where this study was conducted. Samples were however, collected in accordance with the International Union for Conservation of Nature (IUCN) policy statement on research involving species at risk of extinction.

## Results and discussion

### Spectral assessment

**Figure 1:**
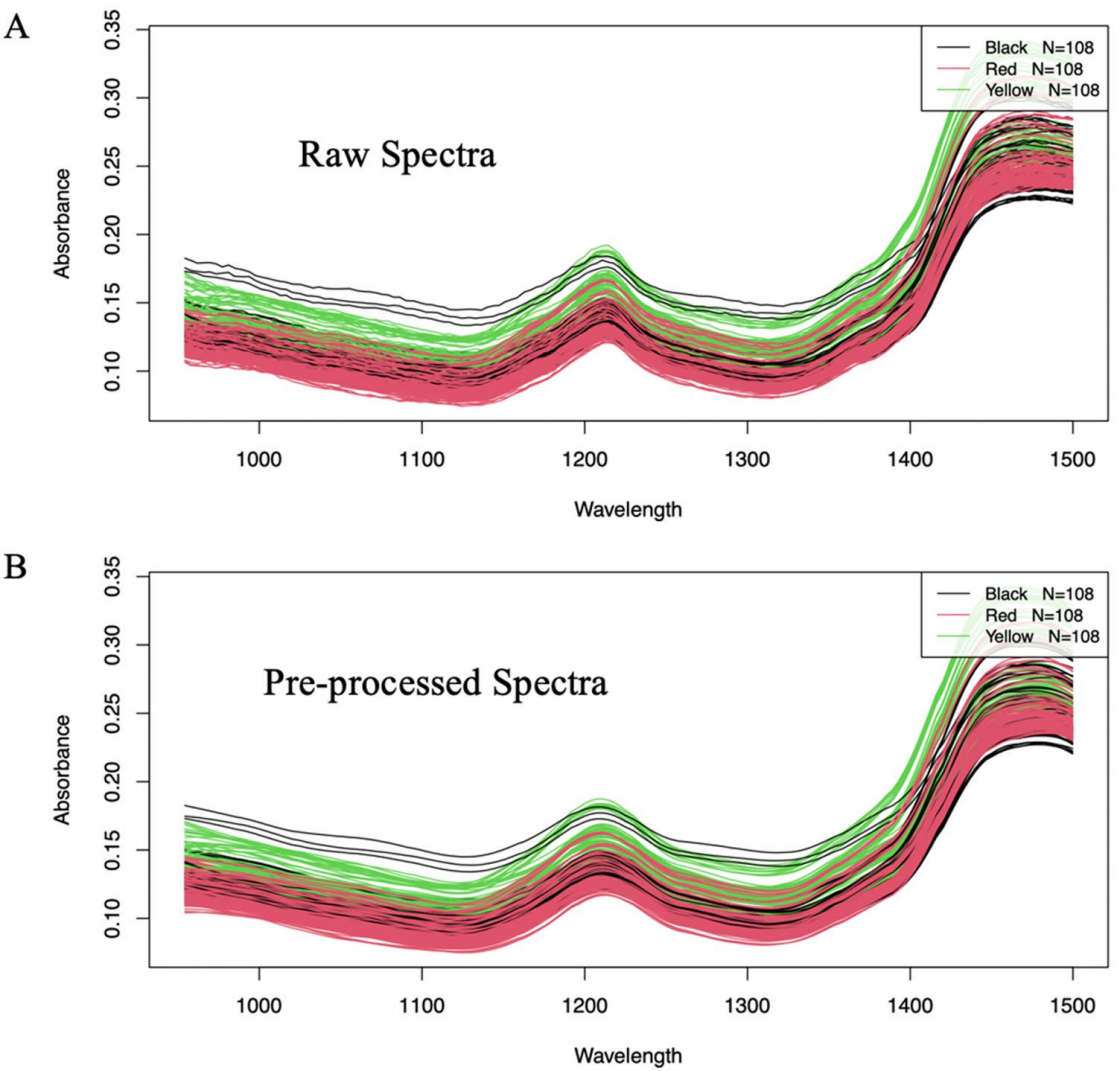
Raw (**A**) and Savitzky-Golay pre-processed and wavelength selected spectra (**B**) plot of yellow, red, and black Maca samples containing 0, 10, 20, 30, 40, and 50% w/w soy and maize.

Figure 1 shows the Raw (A) and Savitzky-Golay pre-processed spectra (B) plot of yellow, red, and black Maca samples containing 0,10, 20, 30, 40, and 50% w/w soy and maize. . From spectral inspection, the extremities of the unprocessed spectra (Fig. 1A) were characterized by noise while the prominent peaks were observed at the wavelength ranges of 950–1500 nm, so this spectral range was selected and used for all subsequent analysis.

**Figure 2:**
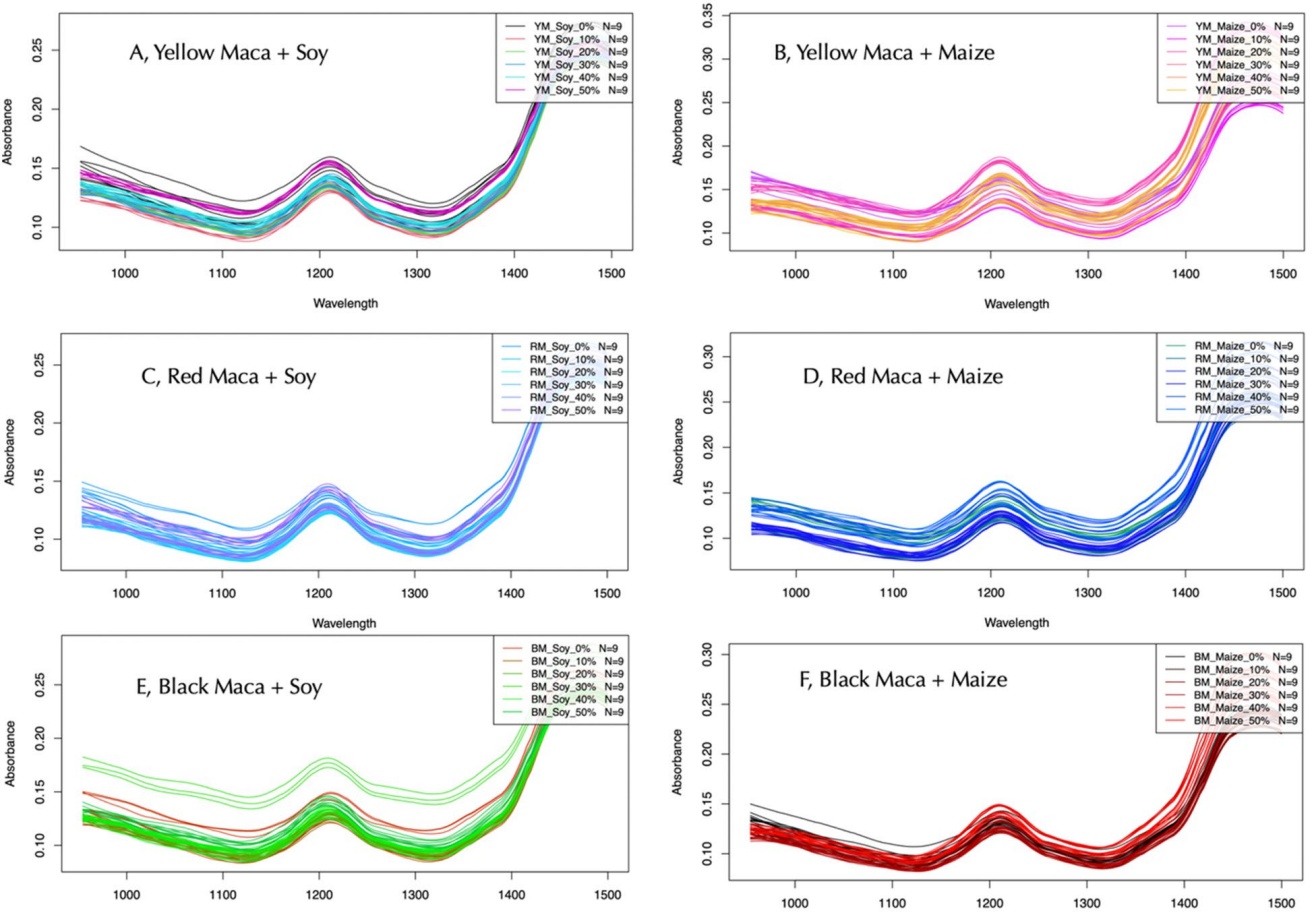
Preprocessed spectra of yellow Maca with soy (**A**), yellow Maca with maize (**B**), Red Maca with soy (**C**), Red Maca with maize (**D**), Black Maca with soy (**E**) and Black Maca with maize (**F**), at different concentrations.

The pre-treated spectra plot of the different forms of maca powder (red, yellow, and black) and the forms of adulterant all presented prominent peaks around 1210 nm and 1450 nm. Based on the absorption characteristics, the Near-infrared wavelength region is generally divided into two regions namely the long-wavelength NIR region (1300–2500 nm), where the absorptions are attributed to the combinations or the first overtones of the O–H (water, alcohol), C-H (fats, oil, hydrocarbons), and N–H (protein) vibrations^23^. The short-wavelength NIR region (700–1300 nm) with absorptions corresponds to the vibration of the second or third overtones which are not as strong and sharp as the former**.** Based on the two prominent peaks observed in the spectral band**.** The resultant peak observed at 1210 nm was due to C − H second overtone and O − H combination associated with C-H bonding, a characteristic group in lipids and proteins, also O − H, N − H stretch first overtone occurs at 1450 nm, which is a characteristic group of moisture^24^**.** Sample absorbance is one of the main topics of interest in spectroscopy, and according to Beer Lamber’s law, the absorbance is directly proportional to the molar absorptivity, sample concentration, and path length^25^**.** The spectra obtained from a sample will be nearly as linear with concentration as transmission spectra. The spectra belonging to the black Maca exhibited the highest absorbance and thus the most transmittance, although it had the least band. The second region which also recorded the most absorbance was the yellow Maca. The red Maca generated a wide band but recorded the least absorbance and thus less transmittance. All three forms of Maca were within the 0.1 to 0.18 absorbance region. The spectral lines of the Maca powder showed that there was a discerning difference among the various Maca types in terms of wavelength and absorbance. The differences obtained in the spectral lines generated by the NIR could be a result of the different particle sizes of the different forms of adulteration the Maca were subjected to^26^. In NIR spectra absorptions of overtones or combinations of fundamental stretching bands occur. The bands, usually caused by C, H, N, or O stretching are weak in intensity and very often overlap^24^. All pre-treated spectra of all three forms of Maca and adulterants have a similar shape (spectra) due to their almost similar chemical structure therefore, it is extremely difficult to distinguish these two compounds with the naked eye although all three forms depicted different bands, and absorbances based on adulterant concentrations. As a result, chemometric analysis is required to overcome such a problem^27^. The preprocessed spectra of yellow maca with soy (A), yellow maca with maize (B), Red maca with soy (C), Red maca with maize (D), Black maca with soy (E) and Black maca with maize (F) , at different concentrations can be found in Fig. 2

### Classification results

#### Classification of red, black, and yellow Maca types

**Figure 3:**
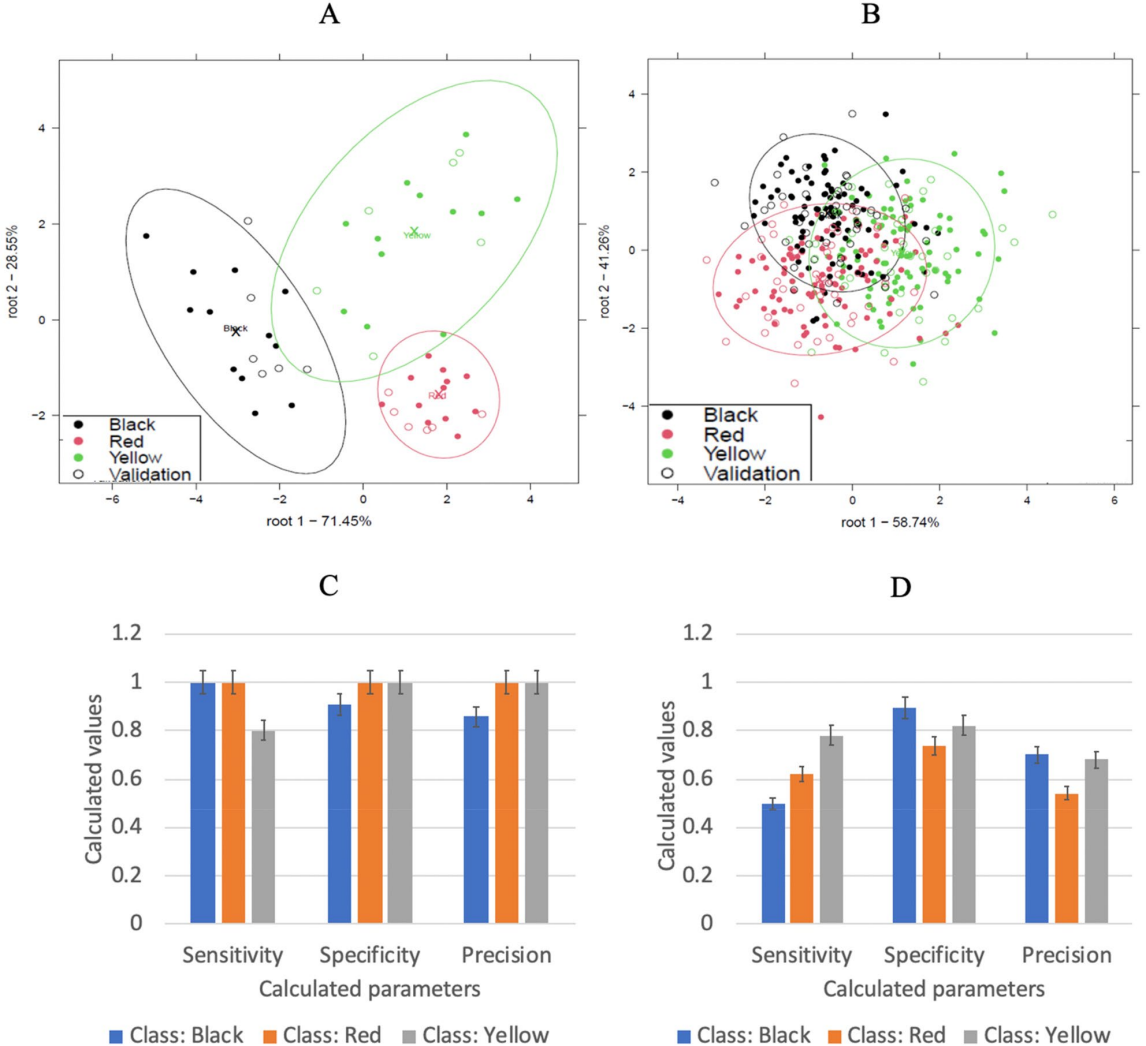
Classification of only pure Yellow, Red, and Black Maca (**A**), classification of Red, and Black containing soy and maize as adulterants (**B**), model performance parameters for the classification of only pure Yellow, Red, and Black maca (**C**) and model performance parameters of Yellow, Red, and Black maca containing soy and maize as adulterants (**D**).

Figure 3 shows the classification of only pure, Yellow, Red, and Black Maca (A), classification of Red, and Black containing soy and maize as adulterants (B), model performance parameters for the classification of only pure Yellow, Red, and Black maca (C) and model performance parameters of Yellow, Red, and Black maca containing soy and maize as adulterants (D).

A more visible pattern of separation could be visualized in Fig. 3(A) than in Fig. 3(B); where it could be observed that there was some overlapping between red, black, and yellow Maca showing no clear distinction between the various Maca colors in Fig. 3(B). Pure red, black, and yellow Maca likely have distinct chemical compositions due to genetic differences etc.. These inherent differences can lead to more pronounced separation in Fig. 3(A) as compared to the adulterated Maca types in Fig. 3(B), which is a mixture of the different maca types and may exhibit intermediate characteristics. Also Adulterated Maca, being a mixture of different types, may exhibit greater variability in its chemical composition compared to pure Maca types. This increased variability can result in less distinct separation between clusters in Fig. 3(B), making it more challenging to discriminate as compared to the pure maca types in Fig. 3(A).This proves that the addition of maize and soy in Maca can lead to challenges of varietal discrimination.

When only pure yellow, red, and black Maca were discriminatedr, there was an average recognition accuracy of 96.38% and prediction accuracy of 94.12% (Fig. 3C). A lower average recognition of 74.10% and an average prediction of 65.53% were obtained for discriminating the different maca varieties containing adulterants (confusion matrices can be viewed in the supplementary sheet Table S2). This suggests that the addition of adulterants influenced not only their genotypic properties but also, their phenotypic property thus, making them less identifiable. Majority of the studies^28–30^ focused on discriminating Maca from different locations, based on their macamide presence and content, using chromatographic and magnetic resonance techniques. The high average prediction and recognition accuracies recorded also prove LDA as robust method in the classification of maca regardless of color, even when adulterants are introduced.

From Fig. 3(C), model performance parameters for the classification of pure maca cultivars were all higher than 0.8 (80%). Although model performance parameters were also higher than 0.5 (50%) when the cultivars were adulterated, it can be observed from Fig. 3(D) that all the performance parameters decreased with the introduction of adulterants. The most affected parameters were the sensitivity of the model for classifying black maca which decreased from 1 (the optimum value) to 0.5 and the precision of the model for classifying red maca. Sensitivity is an absolute quantity, the smallest absolute amount of change that can be detected by a measurement while precision describes the reproducibility of the measurement.

#### Classification of soy and maize in yellow Maca

**Figure 4:**
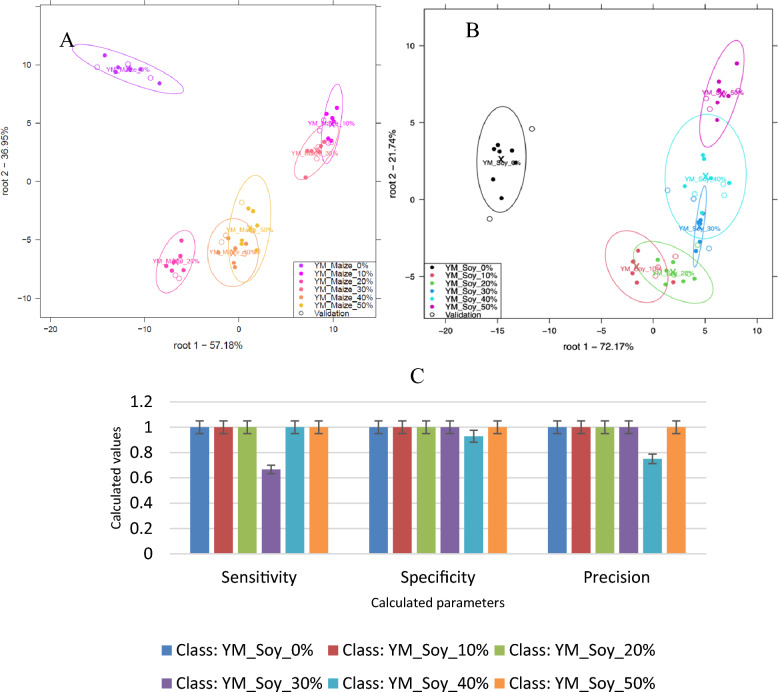
Classification plot for the discrimination of 0, 10, 20, 30, 40, and 50% w/w soy (**A**) and 0, 10, 20, 30, 40, and 50% w/w maize (**B**) in yellow Maca and and model performance parameters for the classification of soy in yellow Maca (**C**).

From Fig. 4(A) and (B), all the different concentration levels were classified and well separated for the discrimination of 0, 10, 20, 30, 40, and 50% w/w soy (Fig. 4A) and 0, 10, 20, 30, 40, and 50% w/w maize (Fig. 4B) in yellow maca although slight overlaps could be observed. In all cases, pure yellow maca was distinctively separated from the adulterated maca samples.

From Fig. 4(C), model performance parameters for the classification of soy in yellow maca powder showed that sensitivity, specificity and precision were all 1 (100%, optimum) except for the classification of 30% adulterated samples which had a sensitivity of 0.67 and 40% adulterated which had a precision of 0.75. Sensitivity, specificity and precision were all 1 (100%, optimum) for the model developed to classify 0, 10, 20, 30, 40, and 50% w/w maize.

Overall, there was an average recognition accuracy of 100% and prediction accuracy of 96.33% for the discrimination of 0,10, 20, 30, 40, and 50% w/w soy in yellow Maca (Fig. 4A). Concentrations, 0, 10, 20, 30, 40, and 50% w/w soy could all be classified with 100% correct accuracy after cross-validation. Confusion matrices can be viewed in the supplementary sheet Table S3.

For the detection of maize adulteration in yellow Maca, there was an overall average recognition accuracy of 100% and prediction accuracy of 95.23% for the discrimination of 0, 10, 20, 30, 40, and 50% w/w maize in yellow Maca (Fig. 4B). All the different concentrations of Maca could be classified with 100% correct accuracy after cross-validation except concentration 40% w/w which was misclassified as concentration 50% w/w. Confusion matrices can be viewed in the supplementary sheet Table S4.

Generally, 0, 10, 20, 30, 40, and 50% w/w maize in yellow Maca could be classified with higher accuracy compared to 0, 10, 20, 30, 40, and 50% w/w soy in yellow Maca. The classification accuracies in the confusion matrixes confirmed the overlaps observed in the plot.

#### Red maca adulteration

**Figure 5:**
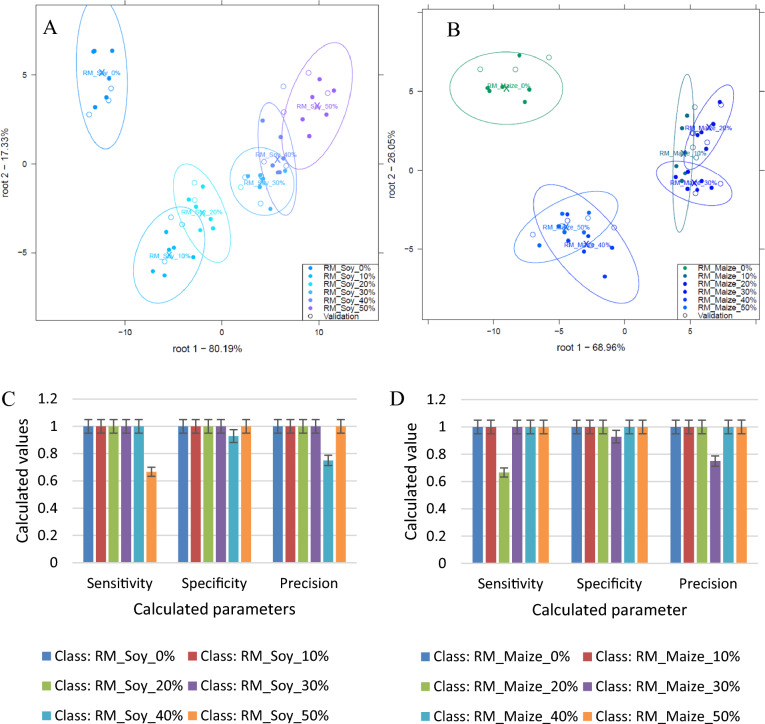
Classification plot for the discrimination of 0, 10, 20, 30, 40, and 50% w/w soy (**A**) and 0, 10, 20, 30, 40, and 50% w/w maize (**B**) in red Maca, model performance parameters for the classification of soy (**C**) and maize (**D**) in red maca.

From Fig. 5, some overlapping could be observed in both plots for the discrimination of 0, 10, 20, 30, 40, and 50% w/w soy (Fig. 5A) and 0, 10, 20, 30, 40, and 50% w/w maize (Fig. 5B) in red Maca. The overlapping was more pronounced in Fig. 5(B) than in Fig. 5(A). Similar chemical constituents between maize and red Maca compositions could lead to overlapping clusters in the PCA plot. If the chemical profiles of maize and red Maca are more similar compared to soy and red Maca, it can result in less distinct separation between the adulterated red Maca with maize concentrations. In all cases, however, pure red Maca was distinctly separated from the adulterated red Maca samples. From Figs. 5(C) and (D), sensitivity, specificity and precision were all above 0.67. Lower concentrations of 0, 10, and 20% w/w/ adulterants had optimum sensitivity, specificity and precision of 1.

Overall, there was an average recognition accuracy of 93.52% and prediction accuracy of 88.94% for the discrimination of 0, 10, 20, 30, 40, and 50% w/w soy in red Maca (Table S5). Only pure red Maca (0% w/w) and 10% w/w soy in red Maca could be classified with 100% correct accuracy after cross-validation. All the other maca concentrations showed misclassifications. Confusion matrices can be viewed in the supplementary sheet Table S5.

For the detection of maize adulteration in red Maca, there was an overall average recognition accuracy of 96.30% and a prediction accuracy of 88.94% for the discrimination of 0, 10, 20, 30, 40, and 50% w/w maize in yellow Maca (Fig. 5B). Thus in average, 88.94% of the instances belonging to each class of concentrations were correctly classified.Only pure red Maca (0% w/w) and 20, 40, and 50% w/w soy in red maca could be classified with 100% correct accuracy after cross-validation. All the other Maca concentrations showed misclassifications. Confusion matrices can be viewed in the supplementary sheet Table S6.

Generally, 0, 10, 20, 30, 40, and 50% w/w maize in red maca could be classified with higher accuracy compared to 0, 10, 20, 30, 40, and 50% w/w soy in red Maca. The misclassification accuracies in the confusion matrixes confirmed the overlapping observed in the plot. And this could be due to the much distinct similarity in the chemical composition of the various Maca types. The high accuracy suggests that the LDA model was effective in distinguishing between the different concentrations of adulterated Maca varieties and making correct predictions.

#### Black maca adulteration

**Figure 6:**
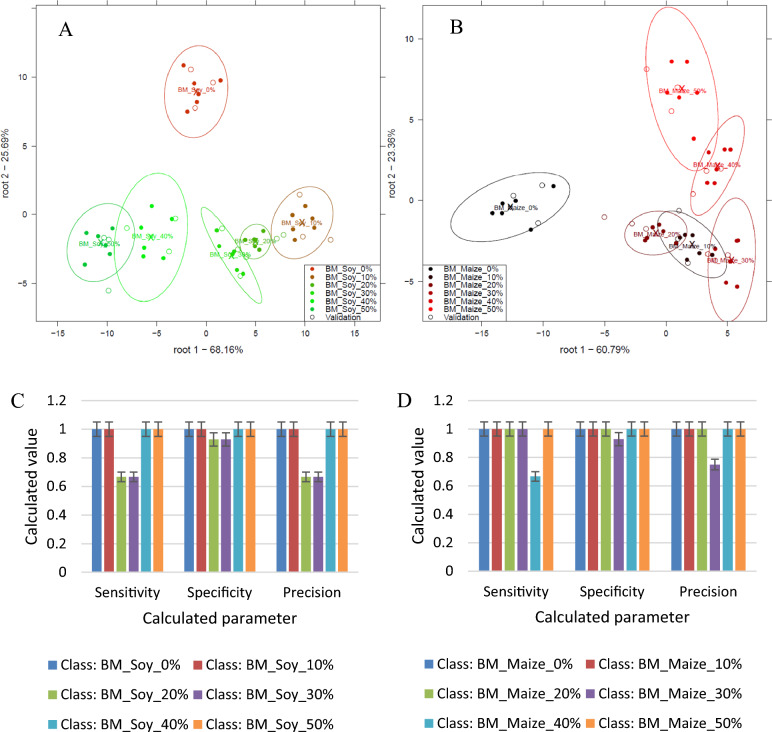
Classification plot for the discrimination of 0, 10, 20, 30, 40, and 50% w/w soy (**A**) and 0, 10, 20, 30, 40, and 50% w/w maize (**B**) in black Maca and model performance parameters for the classification of soy (**C**) and maize (**D**) in black maca.

From Fig. 6(A), all the different concentrations of 0, 10, 20, 30, 40, and 50% w/w soy in black Maca could be distinctively visualized. Some overlapping could, however, be observed in Fig. 6(B) for 0, 10, 20, 30, 40, and 50% w/w maize in black Maca. Overlapping was more pronounced in Fig. 6(B) than in Fig. 6(A). In all cases, however, pure red Maca was distinctly separated from the adulterated red Maca samples. From Fig. 6(C) and (D), sensitivity, specificity and precision were all above 0.67. Better model parameters were achieved for the model developed to classify maize in black than the one developed to classify soy.

Overall, there was an average recognition accuracy of 99.08% and prediction accuracy of 94.44% for the discrimination of 0,10, 20, 30, 40, and 50% w/w soy in red Maca (Fig. 6A). Only pure red Maca (0% w/w) and 10, 40, and 50% w/w soy in red maca could be classified with 100% correct accuracy after cross-validation. Concentrations 20 and 30%w/w soy showed some misclassification. Confusion matrices can be viewed in the supplementary sheet Table S7.

For the detection of maize adulteration in black Maca, there was an overall average recognition accuracy of accuracy of 100% and prediction accuracy of 98.16%. For the discrimination of 0, 10, 20, 30, 40, and 50% w/w maize in yellow Maca (Fig. 6B). Pure black Maca (0% w/w) and all the other concentrations could be classified with 100% correct accuracy after cross-validation except for concentration 40% w/w soy in black Maca. Confusion matrices can be viewed in the supplementary sheet Table S8.

Generally, 0, 10, 20, 30, 40, and 50% w/w maize in black Maca could be classified with higher accuracy compared to 0, 10, 20, 30, 40, and 50% w/w soy in black Maca.

All LDA classification results with adulterants (maize, soybean), regardless of Maca color, show a similar pattern with work by^31^, who coupled NIRS with chemometric techniques in the detection of radish and turnip powder adulteration in Maca. When discriminant analysis was performed, a 100% classification accuracy was achieved in this study. Though the color of the adulterated Maca was not specified, the classification accuracies obtained conferred on NIRS-LDA as very accurate in discriminating pure Maca from adulterated.

### PLSR results of NIRS analysis

#### PLSR results of NIRS analysis on pure maca

Table 1 shows the best pretreatment results which were obtained from the different pretreatment combinations on the raw spectra for the prediction of pure Maca.

**Table 1:** Partial least square regression values obtained from the best pre-treatment combinations on the raw spectra for the prediction of pure maca using leave-one-sample-out cross-validation at a wavelength range of 950–1500 nm.

Preprocessing	R^2^	RMSE(%w/w)	R^2^_CV_	RMSE_CV_(%w/w)	RDP
sgol@2–17-0	0.959	6.273	0.952	6.811	4.602
sgol@2–19-0	0.959	6.277	0.952	6.812	4.602
sgol@2–17-0_snv	0.935	8.160	0.922	8.938	3.613
sgol@2–19-0_snv	0.933	8.227	0.920	8.990	3.557
sgol@2–17-0_msc	0.929	8.414	0.916	9.151	3.459
sgol@2–19-0_msc	0.928	8.428	0.916	9.162	3.455
sgol@2–17-0_deTr	0.948	7.098	0.938	7.771	4.026
sgol@2–19-0_deTr	0.947	7.151	0.937	7.808	3.996
sgol@2–17-0_deTr_snv	0.935	8.015	0.923	8.720	3.588
sgol@2–19-0_deTr_snv	0.934	8.025	0.923	8.716	3.612
sgol@2–17-0_deTr_msc	0.931	8.228	0.920	8.910	3.544
sgol@2–19-0_deTr_msc	0.932	8.219	0.920	8.886	3.554
sgol@2–19-0_sgol@2–19-1	0.944	7.294	0.934	7.940	3.916
sgol@2–19-0_sgol@2–19-2	0.951	7.086	0.939	7.883	4.086
sgol@2–17-0_sgol@2–17-1	0.945	7.228	0.934	7.913	3.918
sgol@2–17-0_sgol@2–17-2	0.949	7.190	0.935	8.109	3.953
sgol@2–19-0_sgol@2–19 1_deTr	0.942	7.654	0.931	8.345	3.816
sgol@2–17-0_sgol@2–17-1_deTr	0.952	7.026	0.943	7.658	4.197

R^2^, R^2^_CV_, Coefficient of determination of model building and validation.

RMSE, RMSE_CV_, Root mean square error of calibration and validation.

**Figure 7:**
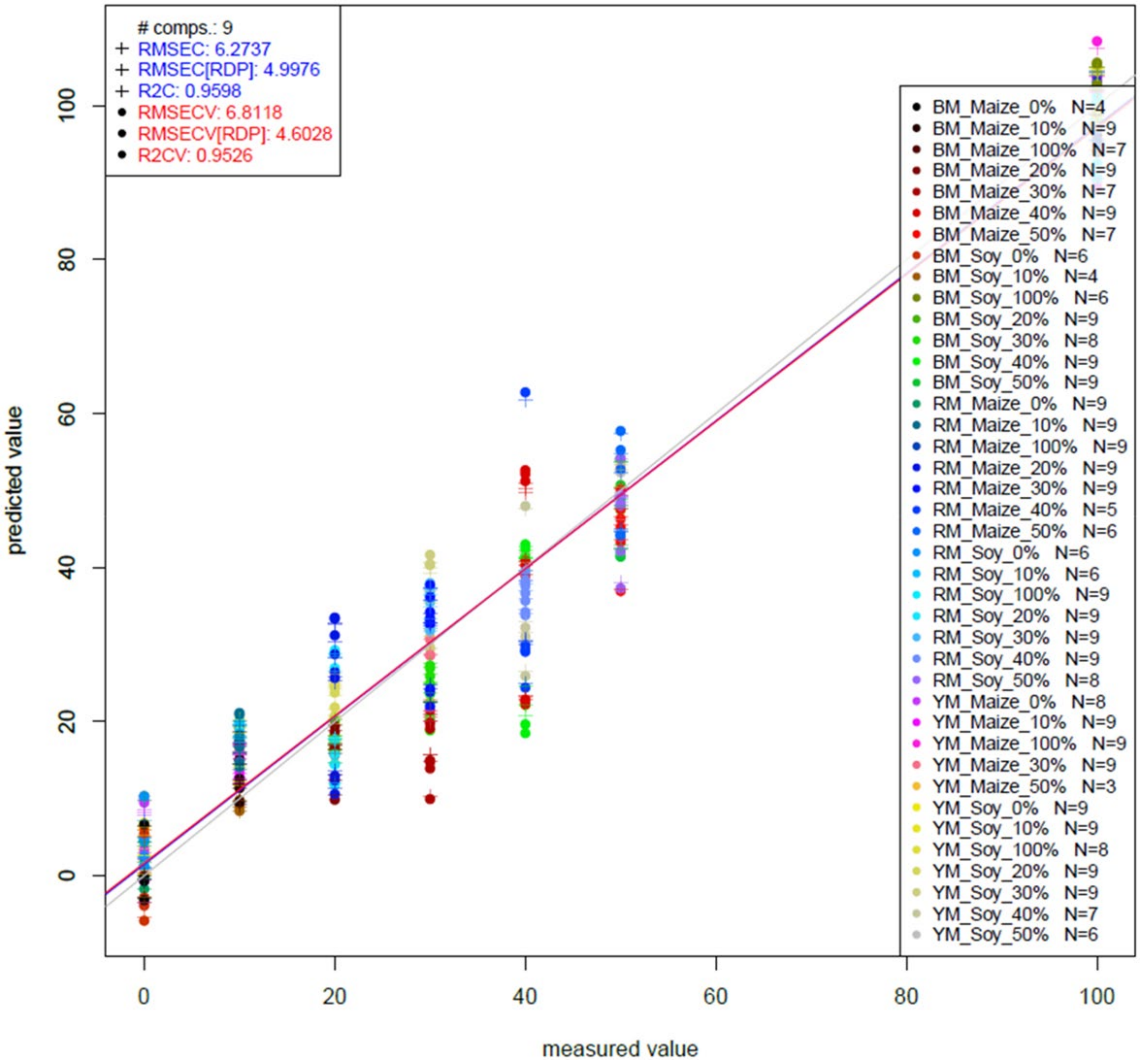
Optimized (Savitzky-Golay smoothing with filter) PLSR plot for predicting soy and maize adulteration in red, yellow, and black Maca powder.

After all the tested 18 pretreatments, Savitzky-Golay smoothing with filters 17 and 19 yielded the best accuracy for predicting pure Maca with an R^2^_CV_ of 0.9528 respectively and RMSE_CV_ of 6.81 w/w pure Maca powder respectively (Fig. 7).

The R^2^ value is a ratio, which represents the coefficient of determination of the calibration model^32^, and is always preferred to be close to 1, which indicates that a good model is produced. The farther it is away from one indicates that is a poor model or not robust. This applies to both the training and cross-validation data sets. RMSE represents the root mean square errors that occur during data processing. The lower the RMSE, the better the model because it indicates the stability of the training and cross-validation model. Higher RMSEs indicate that many errors occurred during data processing and hence the formation of a bad model^33^. RDP represents the ratio of deviation of performance of the model. RDP presents a good model when values are close to 3 or greater^34^. RPDs for all the models in our study were greater than 3. The preferred model was chosen depending on these three main parameters, choosing the pretreatment method that provided the lowest RMSE for both training and cross-validation data while at the same time giving the highest R^2^ value. The model built for predicting and cross-validating Maca adulteration with soy recorded higher precision of R^2^_CV_ and lower RMSE_C_ than that for predicting and cross-validating Maca adulteration with maize. From the different pre-treatments liaised with PLSR, it was easier to detect, predict, and cross-validate adulteration of soy in the various forms of Maca than detecting maize in the three forms of Maca. Overall, the best model which produced the least RMSE_C_, RMSE_CV,_ and RPD while producing the highest R^2^ and R^2^_CV_ was taken into consideration. This was the model achieved with Savitzky-Golay smoothing pretreatment (filters 17 and 19).

## Conclusion

When only pure yellow, red, and black Maca were discriminated against, there was an average recognition accuracy of 96.38% and prediction accuracy of 94.12%. Lower average recognition of 74.10% and an average prediction of 65.53% were obtained for discriminating the different Maca cultivars containing adulterants. From the different pre-treatments liaised with PLSR, it was easier to detect, predict, and cross-validate adulteration of soy in the various forms of Maca than detecting maize in the three forms of Maca. Overall, the best model which produced the least RMSE_C_, RMSE_CV,_ and RPD while producing the highest R^2^ and R^2^_CV_ was taken into consideration. This was the model achieved with Savitzky-Golay smoothing pretreatment (filters 17 and 19). Other pretreatment techniques could also be tested in further studies. The study proved the potential of NIR spectroscopy combined with chemometric analysis for the authenticity and quality control of Maca powder products. Larger sample sizes may be tested for deeper industrial application if required. Plastic bags with properties that are similar to LDPE bags could so be tested in the future.

### Supplementary Information

Supplementary Information.

## Data Availability

The datasets used and/or analysed during the current study available from the corresponding author on reasonable request.

## Code availability

The underlying code for this study [and training/validation datasets] is not publicly available for proprietary reasons.
