# Supplementary material for: Non-invasive prediction of maca powder adulteration using a pocket-sized spectrophotometer and machine learning techniques
Source: Sci Rep. 2024 May 7;14:10426. doi: 10.1038/s41598-024-61220-1 (PMC11076633; doi:10.1038/s41598-024-61220-1)
Supplement: Supplementary file 1 — Supplementary Information. [file 41598_2024_61220_MOESM1_ESM.docx]

Table S1: Spectral preprocessing for partial least squares regression models

| Pretreatment | Action |
| --- | --- |
| sgol@2-17-0 | Savitzky-Golay smoothing with filter 17 |
| sgol@2-19-0 | Savitzky-Golay smoothing with filter 19 |
| sgol@2-17-0_snv | Savitzky-Golay smoothing with filter 17 then standard normal variate |
| sgol@2-19-0_snv | Savitzky-Golay smoothing with filter 19 then standard normal variate |
| sgol@2-17-0_msc | Savitzky-Golay smoothing with filter 17 then multiplicative scatter correction |
| sgol@2-19-0_msc | Savitzky-Golay smoothing with filter 19 then multiplicative scatter correction |
| sgol@2-17-0_deTr | Savitzky-Golay smoothing with filter 17 then detrending |
| sgol@2-19-0_deTr | Savitzky-Golay smoothing with filter 19 then detrending |
| sgol@2-17-0_deTr_snv | Savitzky-Golay smoothing with filter 17 then detrending the standard normal variate |
| sgol@2-19-0_deTr_snv | Savitzky-Golay smoothing with filter 19 then detrending the standard normal variate |
| sgol@2-17-0_deTr_msc | Savitzky-Golay smoothing with filter 17 then detrending then multiplicative scatter correction |
| sgol@2-19-0_deTr_msc | Savitzky-Golay smoothing with filter 19 then detrending then multiplicative scatter correction |
| sgol@2-19-0_sgol@2-19-1 | Savitzky-Golay smoothing with filter 19 then first polynomial derivative fitting |
| sgol@2-19-0_sgol@2-19-2 | Savitzky-Golay smoothing with filter 19 then first polynomial derivative fitting |
| sgol@2-17-0_sgol@2-17-1 | Savitzky-Golay smoothing with filter 17 then first polynomial derivative fitting |
| sgol@2-17-0_sgol@2-17-2 | Savitzky-Golay smoothing with filter 17 then second polynomial derivative fitting |
| sgol@2-19-0_sgol@2-19-1_deTr | Savitzky-Golay smoothing with filter 19 then first polynomial derivative fitting, then detrending |
| sgol@2-17-0_sgol@2-17-1_deTr | Savitzky-Golay smoothing with filter 17 then first polynomial derivative fitting, then detrending |

Table S2: Confusion matrix for the discrimination of Maca types

|  |  |  | Black | Red | Yellow |
| --- | --- | --- | --- | --- | --- |
| Only pure Maca | *Average recognition:*  *96.38%* | Black | 100 | 0 | 2.68 |
|  |  | Red | 0 | 97.25 | 5.43 |
|  |  | Yellow | 0 | 2.75 | 91.89 |
|  | *Average prediction: 94.12%* | Black | 100 | 0 | 11.82 |
|  |  | Red | 0 | 100 | 5.82 |
|  |  | Yellow | 0 | 0 | 82.36 |
| Maca with adulterants at different concentrations | *Average recognition:*  *74.10%* | Black | 75.00 | 13.89 | 10.67 |
|  |  | Red | 14.29 | 70.63 | 12.65 |
|  |  | Yellow | 10.71 | 15.48 | 76.68 |
|  | *Average prediction: 65.53%* | Black | 67.47 | 21.43 | 17.59 |
|  |  | Red | 19.84 | 61.12 | 14.40 |
|  |  | Yellow | 12.69 | 17.45 | 68.00 |

Table S3: Confusion matrix for the discrimination of 0, 10, 20, 30, 40, and 50 % w/w soy in yellow Maca

|  |  | YM_Soy_0% | YM_Soy_10% | YM_Soy_20% | YM_Soy_30% | YM_Soy_40% | YM_Soy_50% |
| --- | --- | --- | --- | --- | --- | --- | --- |
| *Average recognition: 100%* | YM_Soy_0% | 100 | 0 | 0 | 0 | 0 | 0 |
|  | YM_Soy_10% | 0 | 100 | 0 | 0 | 0 | 0 |
|  | YM_Soy_20% | 0 | 0 | 100 | 0 | 0 | 0 |
|  | YM_Soy_30% | 0 | 0 | 0 | 100 | 0 | 0 |
|  | YM_Soy_40% | 0 | 0 | 0 | 0 | 100 | 0 |
|  | YM_Soy_50% | 0 | 0 | 0 | 0 | 0 | 100 |
| *Average prediction: 96.33%* | YM_Soy_0% | 100 | 0 | 0 | 0 | 0 | 0 |
|  | YM_Soy_10% | 0 | 100 | 0 | 0 | 0 | 0 |
|  | YM_Soy_20% | 0 | 0 | 100 | 0 | 0 | 0 |
|  | YM_Soy_30% | 0 | 0 | 0 | 89 | 11 | 0 |
|  | YM_Soy_40% | 0 | 0 | 0 | 11 | 89 | 0 |
|  | YM_Soy_50% | 0 | 0 | 0 | 0 | 0 | 100 |

Table S4: Confusion matrix for the discrimination of 0, 10, 20, 30, 40, and 50 % w/w maize in yellow Maca

|  |  | YM_Maize_0% | YM_Maize_10% | YM_Maize_20% | YM_Maize_30% | YM_Maize_40% | YM_Maize_50% |
| --- | --- | --- | --- | --- | --- | --- | --- |
| *Average recognition: 100%* | YM_Maize_0% | 100 | 0 | 0 | 0 | 0 | 0 |
|  | YM_Maize_10% | 0 | 100 | 0 | 0 | 0 | 0 |
|  | YM_Maize_20% | 0 | 0 | 100 | 0 | 0 | 0 |
|  | YM_Maize_30% | 0 | 0 | 0 | 100 | 0 | 0 |
|  | YM_Maize_40% | 0 | 0 | 0 | 0 | 100 | 0 |
|  | YM_Maize_50% | 0 | 0 | 0 | 0 | 0 | 100 |
| *Average prediction: 98.16%* | YM_Maize_0% | 100 | 0 | 0 | 0 | 0 | 0 |
|  | YM_Maize_10% | 0 | 100 | 0 | 0 | 0 | 0 |
|  | YM_Maize_20% | 0 | 0 | 100 | 0 | 0 | 0 |
|  | YM_Maize_30% | 0 | 0 | 0 | 100 | 0 | 0 |
|  | YM_Maize_40% | 0 | 0 | 0 | 0 | 89 | 0 |
|  | YM_Maize_50% | 0 | 0 | 0 | 0 | 11 | 100 |

Table S5: Confusion matrix for the discrimination of 0, 10, 20, 30, 40, and 50 % w/w soy in red Maca

|  |  | RM_Soy_0% | RM_Soy_10% | RM_Soy_20% | RM_Soy_30% | RM_Soy_40% | RM_Soy_50% |
| --- | --- | --- | --- | --- | --- | --- | --- |
| *Average recognition: 93.52%* | RM_Soy_0% | 100 | 0 | 0 | 0 | 0 | 0 |
|  | RM_Soy_10% | 0 | 100 | 0 | 0 | 0 | 0 |
|  | RM_Soy_20% | 0 | 0 | 94.5 | 0 | 0 | 0 |
|  | RM_Soy_30% | 0 | 0 | 5.5 | 94.5 | 11.17 | 0 |
|  | RM_Soy_40% | 0 | 0 | 0 | 5.5 | 88.83 | 16.67 |
|  | RM_Soy_50% | 0 | 0 | 0 | 0 | 0 | 83.33 |
| *Average prediction: 88.94%* | RM_Soy_0% | 100 | 0 | 0 | 0 | 0 | 0 |
|  | RM_Soy_10% | 0 | 100 | 0 | 0 | 0 | 0 |
|  | RM_Soy_20% | 0 | 0 | 89 | 0 | 0 | 0 |
|  | RM_Soy_30% | 0 | 0 | 11 | 89 | 11 | 0 |
|  | RM_Soy_40% | 0 | 0 | 0 | 11 | 89 | 33.33 |
|  | RM_Soy_50% | 0 | 0 | 0 | 0 | 0 | 66.67 |

Table S6: Confusion matrix for the discrimination of 0, 10, 20, 30, 40, and 50 % w/w maize in red Ma

|  |  | RM_Maize_0% | RM_Maize_10% | RM_Maize_20% | RM_Maize_30% | RM_Maize_40% | RM_Maize_50% |
| --- | --- | --- | --- | --- | --- | --- | --- |
| *Average recognition: 96.30%* | RM_Maize_0% | 100 | 0 | 0 | 0 | 0 | 0 |
|  | RM_Maize_10% | 0 | 83.33 | 0 | 0 | 0 | 0 |
|  | RM_Maize_20% | 0 | 16.67 | 100 | 5.5 | 0 | 0 |
|  | RM_Maize_30% | 0 | 0 | 0 | 94.5 | 0 | 0 |
|  | RM_Maize_40% | 0 | 0 | 0 | 0 | 100 | 0 |
|  | RM_Maize_50% | 0 | 0 | 0 | 0 | 0 | 100 |
| *Average prediction: 88.94%* | RM_Maize_0% | 100 | 0 | 0 | 0 | 0 | 0 |
|  | RM_Maize_10% | 0 | 77.67 | 0 | 0 | 0 | 0 |
|  | RM_Maize_20% | 0 | 22.33 | 89 | 11 | 0 | 0 |
|  | RM_Maize_30% | 0 | 0 | 11 | 89 | 0 | 0 |
|  | RM_Maize_40% | 0 | 0 | 0 | 0 | 89 | 11 |
|  | RM_Maize_50% | 0 | 0 | 0 | 0 | 11 | 89 |

Table S7: Confusion matrix for the discrimination of 0, 10, 20, 30, 40, and 50 % w/w soy in black maca

|  |  | BM_Soy_0% | BM_Soy_10% | BM_Soy_20% | BM_Soy_30% | BM_Soy_40% | BM_Soy_50% |
| --- | --- | --- | --- | --- | --- | --- | --- |
| *Average recognition: 99.08%* | BM_Soy_0% | 100 | 0 | 0 | 0 | 0 | 0 |
|  | BM_Soy_10% | 0 | 100 | 0 | 0 | 0 | 0 |
|  | BM_Soy_20% | 0 | 0 | 100 | 5.5 | 0 | 0 |
|  | BM_Soy_30% | 0 | 0 | 0 | 94.5 | 0 | 0 |
|  | BM_Soy_40% | 0 | 0 | 0 | 0 | 100 | 0 |
|  | BM_Soy_50% | 0 | 0 | 0 | 0 | 0 | 100 |
| *Average prediction: 94.44%* | BM_Soy_0% | 100 | 0 | 0 | 0 | 0 | 0 |
|  | BM_Soy_10% | 0 | 100 | 0 | 0 | 0 | 0 |
|  | BM_Soy_20% | 0 | 0 | 89 | 22.33 | 0 | 0 |
|  | BM_Soy_30% | 0 | 0 | 11 | 77.67 | 0 | 0 |
|  | BM_Soy_40% | 0 | 0 | 0 | 0 | 100 | 0 |
|  | BM_Soy_50% | 0 | 0 | 0 | 0 | 0 | 100 |

Table S8: Confusion matrix for the discrimination of 0, 10, 20, 30, 40, and 50 % w/w maize in black Maca

|  |  | BM_Maize_0% | BM_Maize_10% | BM_Maize_20% | BM_Maize_30% | BM_Maize_40% | BM_Maize_50% |
| --- | --- | --- | --- | --- | --- | --- | --- |
| *Average recognition: 100%* | BM_Maize_0% | 100 | 0 | 0 | 0 | 0 | 0 |
|  | BM_Maize_10% | 0 | 100 | 0 | 0 | 0 | 0 |
|  | BM_Maize_20% | 0 | 0 | 100 | 0 | 0 | 0 |
|  | BM_Maize_30% | 0 | 0 | 0 | 100 | 0 | 0 |
|  | BM_Maize_40% | 0 | 0 | 0 | 0 | 100 | 0 |
|  | BM_Maize_50% | 0 | 0 | 0 | 0 | 0 | 100 |
| *Average prediction: 98.16%* | BM_Maize_0% | 100 | 0 | 0 | 0 | 0 | 0 |
|  | BM_Maize_10% | 0 | 100 | 0 | 0 | 0 | 0 |
|  | BM_Maize_20% | 0 | 0 | 100 | 0 | 0 | 0 |
|  | BM_Maize_30% | 0 | 0 | 0 | 100 | 11 | 0 |
|  | BM_Maize_40% | 0 | 0 | 0 | 0 | 89 | 0 |
|  | BM_Maize_50% | 0 | 0 | 0 | 0 | 0 | 100 |
